# Small‐Scale Stabilizing Effect of Asynchrony in Vital Rate Responses to Climate in an Imperiled Dune Thistle

**DOI:** 10.1002/ece3.72080

**Published:** 2025-08-31

**Authors:** E. Binney Girdler, Tiffany M. Knight, Sanne M. Evers, Aldo Compagnoni, Roxanne Leberger, Julie E. Marik, Samara I. Hamzé, Claudia L. Jolls

**Affiliations:** ^1^ Department of Biology Kalamazoo College Kalamazoo Michigan USA; ^2^ University of Michigan Biological Station Pellston Michigan USA; ^3^ Department of Community Ecology Helmholtz Centre for Environmental Research—UFZ Halle (Saale) Germany; ^4^ German Centre for Integrative Biodiversity Research (iDiv) Halle‐Jena‐Leipzig Leipzig Germany; ^5^ Institute of Biology Martin Luther University Halle‐Wittenberg Halle (Saale) Germany; ^6^ Department of Biology East Carolina University Greenville North Carolina USA

**Keywords:** climate drivers, coastal dunes, spatial variation, thistle, vital rates

## Abstract

Changes in population responses to climate are usually studied at broad spatial grains, such as across species ranges. Only a handful of studies have investigated how small‐scale variation, for example driven by soil conditions and microtopography, can mediate the responses of population vital rates to climate. Here, we examine responses of *Cirsium pitcheri* vital rates to climate across five subpopulations occurring in coastal dune locations that range from the foredune to the backdune. We consider five vital rates: seedling survival, adult survival, growth, flowering probability, and the size of new adults. We use generalized linear mixed models to quantify vital rate variation across the dune gradient. Then, we analyze the estimates to quantify the so‐called “portfolio effect”: the stabilizing effect of spatial asynchrony among the five subpopulations on the population as a whole. Finally, we use a sliding window approach to test whether climate drivers contribute to the portfolio effect. We found evidence of substantial portfolio effects across the dune gradient. These effects arise in part from the opposite responses of vital rates to climatic drivers in our five subpopulations. Strikingly, seedling survival, adult survival, and growth were negatively correlated with precipitation in the subpopulation closer to shore and positively correlated with precipitation in the subpopulation farthest from the shore. We explain this pattern by noting that storms that bring precipitation to the dune system may also cause disturbance and sand burial that negatively affect plants close to the water's edge. Such climate‐mediated portfolio effects should have a stabilizing effect on the population abundance over time, with implications for population projections and for assessments of range‐wide vulnerability of rare plant species. We call for further scrutiny of variation in small‐scale responses to climate and for further empirical tests, especially in coastal dune environments.

## Graphical abstract

We examined climate‐mediated portfolio effects within a population of the coastal dune endemic plant *Cirsium pitcheri* . We found that vital rates responded in opposite directions to climatic drivers, especially precipitation, in our five subpopulations that spanned the foredune to backdune. Such asynchronous responses at small spatial scales could have a stabilizing effect on the population abundance over time, with implications for population projections and for assessments of range‐wide vulnerability of rare plant species.

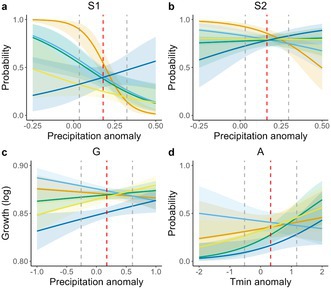

## 1 Introduction

Understanding how the demographic vital rates (survivorship, growth, fecundity) of plants and animals respond to climate is important for predicting how populations will be affected by climate change (Bellard et al. 2012; Urban et al. 2016). Responses of plant and animal populations to climate drivers are known to depend on the environmental context (Patsiou et al. 2014; Peery et al. 2012). Understanding the relationships between demography, climate, and environment is critical for developing strategies to manage rare species and biodiversity in the face of climate change.

While there is much research on how population responses to climate vary across species' ranges, far fewer studies examine such differences at smaller spatial scales. Plant species' responses to climate can depend on local conditions, such as soil (Lindell et al. 2022; Nicolè et al. 2011) and topography (Ackerly et al. 2020; Patsiou et al. 2014). In animals, studies have found spatial heterogeneity in forest structure impacts climate effects on birds (Betts et al. 2018) and thermal microclimate variation in urban versus rural habitats can be considerable for ectotherms (Pincebourde et al. 2016). Such small‐scale environmental variation may affect population dynamics and determine the persistence of populations via the portfolio effect (PE). A PE occurs when subpopulations of the same species vary asynchronously. Such asynchronous variation dampens the variability in the abundance of the entire population—similarly to how, in the study of finance, a diversified investment portfolio decreases risk (Schindler et al. 2015). The strength of the PE reflects the correlation in the variation of subpopulations, being zero when correlation is 1 (perfect synchrony) and maximum when correlation is −1 (perfect asynchrony). Such portfolio effects have been observed to stabilize population growth in both plants and animals (Schindler et al. 2010; Abbott, Doak, and DeMarche 2017; Dibner et al. 2019; Oldfather and Ackerly 2018). For example, in plants, portfolio effects have been shown to stabilize the response of populations to climate in populations across 5 km^2^ (Dibner et al. 2019) and 5 ha (Abbott, Doak, and DeMarche 2017).

Climate can contribute to portfolio effects, but successfully linking climate to vital rates is complicated by at least two factors: the need for long‐term data and the need to identify the appropriate time window during which vital rates respond to climate. Most studies make a priori choices that growing season climate or annual climate should best predict the vital rates in a given year. However, such choices are at odds with literature showing that species can have lagged responses to climate (Evers et al. 2021). For example, individuals can take more than a year to die after suffering from drought stress (Bigler et al. 2007; Klockow et al. 2018) or respond strongly to the climate of the dormant or cold season (Czachura and Miller 2020; Hindle et al. 2019). Thus, overlooking the time window during which climate has the largest influence on vital rates could hinder progress in scientific understanding and management (Evers et al. 2023). Fortunately, there is growing evidence that statistical techniques such as the sliding window approach can successfully select the time window(s) during which a climate variable best predicts vital rates (e.g., Brommer et al. 2008; Evers et al. 2021; Husby et al. 2010; van de Pol et al. 2016).

In this study, we analyze a demographic dataset that is both long term (17 years) and encompasses small‐scale spatial variation across a well‐known gradient. *Cirsium pitcheri* is a threatened plant species endemic to Laurentian Great Lakes dune ecosystems. Previous work (Halsey et al. 2016) has demonstrated that *C. pitcheri* populations differ in which demographic vital rate contributes most to population growth. These differences were tied to population age and habitat factors such as successional stage, as also found for other plant species (Burns et al. 2013). While spatial variation between *C. pitcheri* subpopulations has been studied (Halsey et al. 2016; Nantel et al. 2018), responses of subpopulations to climate drivers have not (but see Rand et al. 2015; Rivera et al. 2021).

Foredunes and backdunes differ in many environmental factors, including moisture availability, wind exposure, and soil composition (Doing 1985; Maun and Perumal 1999). The spatial location on the dune might also determine the relationship between climate‐related disturbance or stress and demography. To the best of our knowledge, no study has tested how climate–vital rate relationships vary across subpopulations of plants in different dune locations that range from the foredune to the backdune.

Here, we use three analyses to investigate the variation in demographic rates of *Cirsium pitcheri* across time (17 years) and space (five zones spanning foredune to backdune). First, we fit generalized linear mixed models designed to quantify how vital rates and population growth vary based on year and zone. Second, we use these data to quantify the strength of the PE by examining the synchrony of population numbers, vital rates, and population growth rate across zones. Finally, we investigate whether climate contributes to the PE by testing for a climate‐by‐zone interaction using a sliding windows approach (van de Pol et al. 2016). Our ultimate goal was to investigate whether climate‐by‐zone interactions contribute to a small‐scale stabilizing PE in *C. pitcheri* .

## 2 Methods

### 2.1 Study Species

**FIGURE 1:**
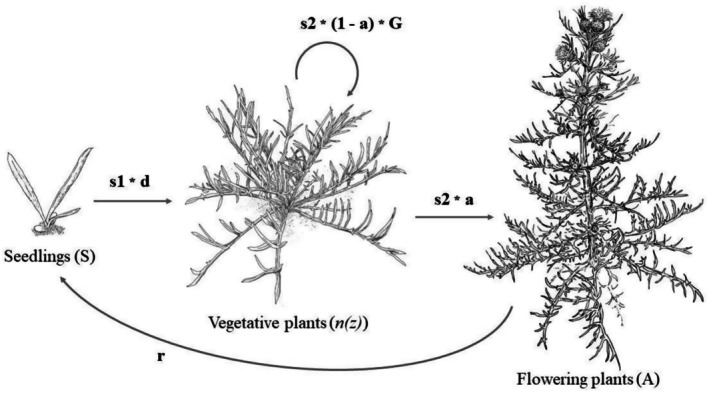
Demographic monitoring of *Cirsium pitcheri* tracks three life stages. Seedlings were tagged but not measured for size; vegetative rosettes were measured for size as taproot diameter just below the crown of leaves; flowering plants were not measured and die the year they flower in this monocarpic species. Life cycle diagram to illustrate the vital rates modeled in the integral projection model. *s*1: Seedling survivorship, *d*: New plant size; *s*2: Plant survivorship; *G*: Growth; *a*: Probability of flowering; *r*: Recruitment (seedlings per reproductive plant). Drawings by E. Binney Girdler.

*Cirsium pitcheri* (Pitcher's thistle) is a USA federally listed threatened native plant, narrowly endemic to the western Great Lakes dunes, where it colonizes open sandy areas maintained by cyclic natural disturbance processes (Loveless 1984; McEachern et al. 1994). Plants live 3–12 years as nonflowering vegetative rosettes, then flower once, and die (Figure 1). This species has been considered to have a metapopulation structure in dynamic dune systems dominated by natural disturbance and sand movement via wind and wave action (Halsey et al. 2016; McEachern 1992; McEachern et al. 1994; Rowland and Maun 2001). Populations of *C. pitcheri* exhibit large yearly fluctuations in density, suggested to be due to variation in rates of seedling establishment and sizes of juvenile plants (McEachern 1992), number of plants flowering (D'Ulisse and Maun 1996), herbivory (Nantel et al. 2018; Rowland and Maun 2001), and shoreline erosion (Nantel et al. 2018).

### 2.2 Field Data Collection

**FIGURE 2:**
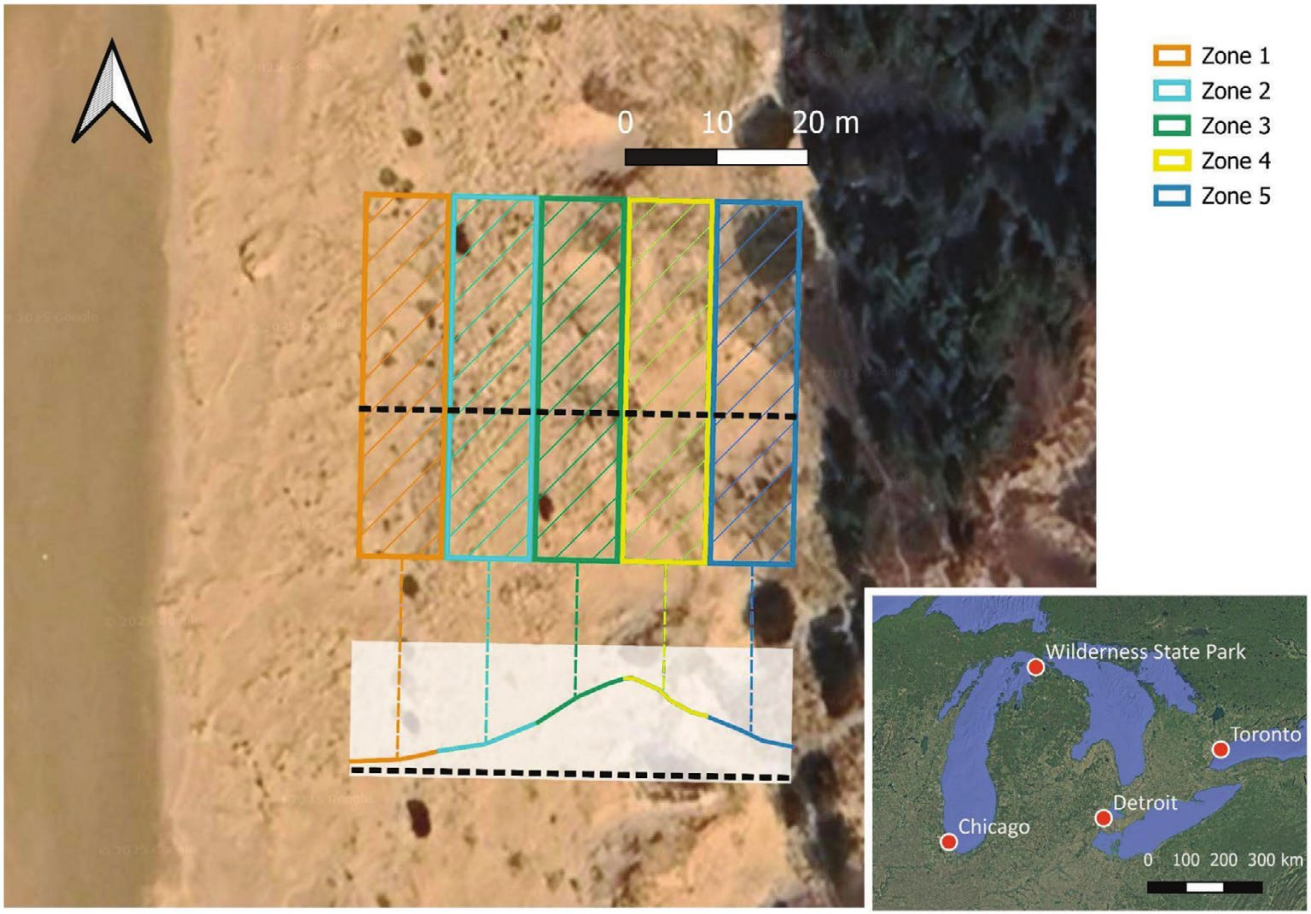
Demographic monitoring plot at Wilderness State Park, Michigan, USA (45.72°, −84.94°); inset shows location within the Great Lakes and the location of Chicago, Detroit, and Toronto. Zones are 10 m × 40 m oriented with the long axis parallel to the lake shore. Symbology is consistent within subsequent figures. Zones 1, 2, and 3 have a west‐facing (lakeward) aspect and increase in elevation with distance from the lake, with Zone 3 having the steepest slope. Zones 4 and 5 have an east‐facing (inland) aspect and decrease in elevation, with Zone 4 having a steeper slope than Zone 5. The elevation profile depicts the estimated qualitative change in elevation from the shore to the forest; we do not have quantitative elevation data for the site, although GPS records provide rough estimates of vertical scale (m asl = meters above sea level) spanning 3 m in elevation from the toe to the top of the dune. Lake level for the 2025 satellite photo from Google Earth was approximately 176 m a.s.l.; the water line is approximately 50 m west of the plot.

Demographic data have been collected annually from 2006 to 2022 in a plot located on the shores of Sturgeon Bay on Lake Michigan, Wilderness State Park, Emmet Co., MI, USA (45.72°, −84.94°, Figure 2). Our 40 m × 50 m plot was established to monitor the demography of this endemic rare species (Havens et al. 2012; Jolls et al. 2015). The plot was designed to span the dune gradient from the lake shoreline to the treeline in the 50 m direction, and the 40 m along‐shore distance resulted in a manageable size to census in a 1‐week period. This 2000 m^2^ plot size comprised hundreds of plants needed for demographic analysis. The plot includes a gradient of elevation typical of linear dune systems in the Great Lakes and elsewhere, including foredune and backdune. We divided our plot into five zones from the shoreline inland that captured spatial variation not quantified directly, for example, aspect (Zones 1, 2, and 3 face west; Zones 4 and 5 face east), slope (Zones 3 and 4 are steepest), and vegetative cover (Zones 1 and 2 are more sparse). Because of the north–south orientation of the dune ridge, variation in insolation due to southern versus northern exposure, although undoubtedly present at a very small scale, is not a distinguishing factor between zones. Zones 1–5 represent a progression from lake to forest (west to east at this site, Figure 2).

Individuals were followed through time using numbered ID stamps on metal tags affixed to *C. pitcheri* plants with vinyl‐coated stainless steel wire. In late June or early July each year, the plot was swept with a metal detector to locate previously tagged individuals and tags from dead individuals. New seedlings received new tags but were not measured to avoid potential damage. Plant size was measured as taproot diameter just below the crown on nonflowering *C. pitcheri* plants. If an individual was not found for three or more years, we scored the individual as dead in the first year it went missing (on a few occasions, we found a plant after missing it in previous years; after 3 years, we could be more confident it was dead even if we did not recover the tag). Population abundance for each census year included all live, tagged plants (seedlings, vegetative plants, and reproductive plants, Figure 1) recorded in each of the five zones (Figure 2).

### 2.3 Variation in Vital Rates

We used generalized linear mixed models to quantify the variation in *C. pitcheri* vital rates across zones and years. We considered five different vital rates: seedling and vegetative (nonreproductive) plant survival (S1 and S2, respectively) from year *t*0 to year *t*1, changes in size of nonreproductive plants (i.e., growth, G) from year *t*0 to year *t*1, the probability that nonreproductive plants in year *t*0 become flowering adults (adults, A) in year *t*1, and the size of new vegetative plants (D, size of a plant the year after it was a seedling as diameter of the taproot measured with calipers). We modeled S1 and D as size‐independent (seedlings were not measured, and thus we did not consider how seedling size might influence seedling survivorship or the size of the plant in the next year). We assume the main predictor of S2, G, and A is size, represented here by the natural logarithm of taproot diameter. We modeled S1, S2, and A as a Bernoulli‐distributed process using a logit link and D and G each as a normally distributed variable.

To quantify the potentially different vital rate responses of plants in different zones to different years, we fit and plotted models using a random effect of the year‐by‐zone interaction only. We chose this model structure to emphasize the variation across years and zones. This objective would have been hindered by fitting models that simultaneously include random effects of year, zone, and year‐by‐zone interaction. Such models would attenuate the differences among zones fluctuating in opposite directions within the same year. For instance, the survival of plants in Zone 1 and Zone 5 might fluctuate in opposite directions going from 2016 to 2017. If this were true, a model with independent year and zone terms would bias estimates toward the mean and the respective year and zones, minimizing the asynchrony between zones reflected by model estimates. In the Supporting Information, we show several alternatives to plot these data (Figures S1 and S2). Finally, we also show in Figure S3 how size affected S2, G, and A in each of the five zones by plotting mean model predictions across all observed sizes for a single year (2018).

### 2.4 Population Growth Rate Across Zones and Years

We utilized an integral projection model (IPM, Ellner et al. 2016) to estimate the asymptotic growth rate (*λ*) of populations across zones and years. We used *λ* as it reflects the effect of variation in vital rates on the change in population numbers (Ellis and Crone 2013). This life cycle is divided into three stages (Figure 1): seedlings (*S*), nonreproductive plants (*n*(*z*)), and reproductive individuals (*A*). Only the number of nonreproductive plants is structured by size (*z*). The change in the number of nonreproductive plants of size z in year t, from 1 year (*n*(*z*, *t*0)) to the number of nonreproductive plants of size *z*′ in year *t* + 1 (*n*(*z′*, *t*1)) is delineated by:

(1) $$n\left(z^{\prime },t1\right)=S\left(t0\right)s1d\left(z^{\prime }\right)+\int_{L}^{U}s2\left(z\right)\left(1-a(z\right))G\left(z^{\prime },z\right)n\left(z,t0\right)\mathrm{dz}$$

The first term accounts for seedlings entering the nonreproductive class, where *S*(*t*0) is the number of seedlings at time *t*0, *s*1 is seedling survivorship, and *d*(*z*') is the size distribution of new nonreproductive plants at time *t*1. The second term accounts for the nonreproductive plants that persist in the same class from *t*0 to *t*1, defined as an integral whose upper and lower limits are, respectively, *U* and *L*. These are the sizes of the largest and smallest *C. pitcheri* observed during the census. This number depends on the size‐dependent adult survival probability, *s*2(*z*), on the size‐dependent probability of not flowering, (1*‐a*(*z*)), and on the changes from size *z* to *z*′, presented as *G*(*z*′,*z*).

The number of seedlings in the population at time *t*1, *S*(*t*1), is the product of *A*(*t*0), the number of reproductive adults at time *t*0, and *r*, the number of seedlings produced per reproductive adult:

(2) $$S\left(t1\right)=A\left(t0\right)r$$

The number of adults at time *t*1 is described by the formula:

(3) $$A\left(t1\right)=\int_{L}^{U}s2\left(z\right)a\left(z\right)n\left(z,t0\right)\mathrm{dz}$$

Note that every adult dies after reproduction. We implemented the integrals using the midpoint rule over 200 equally spaced size bins. To account for unintentional eviction, we used truncated probability distributions, and we extended the *L* and *U* values to 20% of the observed minimum and maximum sizes. Year‐ and zone‐specific vital rates were modeled as described above for S1, S2, G, D, and A. Recruitment, *r*, was zone‐specific and estimated directly from the data as the number of seedlings in year *t*1 divided by the number of reproductive adults in year *t*0. We pooled across years to avoid dividing by zero; we saw cases of zones having seedlings present in year *t*1 even though there were no flowering plants in year *t*0. These seedlings could represent either a seedbank or between‐zone dispersal; we did not have enough data to discriminate between the two. *Cirsium pitcheri* has been found to have a small seedbank (Hamzé and Jolls 2000) that is unlikely to contribute to population persistence (USFWS 2002). Although we did have information on the number of capitula per flowering plant, this information was no more predictive of next year's seedlings than knowing the number of plants in a zone. Therefore, for the IPM, we used the more parsimonious option. Using the *ipmr* R package (Levin et al. 2021) to implement the IPM, we computed the asymptotic population growth rate (*λ*) for each zone and year by iterating the IPMs until the population growth rate converged.

### 2.5 Portfolio Effects

Because portfolio effects are driven by spatial asynchrony, we first quantified the asynchrony of each vital rate and of the population growth rates (*λ*). We quantified asynchrony by computing the pairwise correlation coefficients of the vital rates and population growth rates (*λ*) among the five zones. For the vital rates, we performed this analysis on the mean zone‐ and year‐specific predictions of the generalized linear mixed models (see “Variation in vital rates” above). For the population growth rates (*λ*), we used the zone‐ and year‐specific *λ* values produced by the IPM. For all metrics, we computed correlation matrices and tested the significance of correlations using function cor.test().

To quantify the PE in our population, and to test whether climate had a role in it, we ran two separate analyses. First, we quantified the total PE using our abundance data to calculate the population‐level synchrony index (*φ*, Loreau and de Mazancourt 2008; Thibaut and Connolly 2013), and the strength of the PE (PE, Anderson et al. 2013). The population‐level synchrony index is calculated as

(4) $$\varphi =\frac{\mathrm{Var}\left(N_{\text{total}}\right)}{{\left(\sum_{i}\sqrt{\mathrm{Var}\left(N_{i}\right)}\right)}^{2}}$$

where the numerator is the yearly variance in the abundance of the whole population, *N*_total_, and in the denominator, Var(*N*_*i*_) refers to the variation in abundance of zone *i*. This synchrony index varies from 0 to 1, with values closer to 0 reflecting perfectly asynchronous populations and therefore a stronger portfolio effect. We calculated the PE using the two‐step procedure described in Anderson et al. (2013). First, we estimate the temporal mean–variance scaling relationship, *z*, by fitting the linear regression.

(5) $$\log\;\left(\;\mathrm{Var}\left(N_{i}\right)\right)=\beta_{0}+z\log\left(E\left(N_{i}\right)\right)+\varepsilon_{i}$$

where subscript *i* refers to one of our five zones (as in Equation 4), *E* is the expectation operator, $\beta_{0}$ is an intercept, and $\varepsilon_{i}$ represents the normally distributed residual variance. We use $\beta_{0}$ and *z* to compute the variance predicted for the temporal mean of the abundance of the total population,

(6) $$\mathrm{Var}{\left(N_{\text{total}}\right)}_{\text{Pred}}=e^{\beta_{0}+z\log\left(E\left(N_{\text{total}}\right)\right)}$$

Finally, we calculate the PE as.

(7) $$\mathrm{PE}=\frac{\mathrm{Var}{\left(N_{\text{total}}\right)}_{\text{Pred}}}{\mathrm{Var}{\left(N_{\text{total}}\right)}_{\mathrm{Obs}}}$$

where the denominator is simply the yearly variance in the total number of individuals observed in the data. PE values above 1 are evidence for asynchrony, and therefore, portfolio effects.

### 2.6 Climate Data

For the climate data used in the sliding window analysis, we used monthly data from the closest NOAA (National Oceanic and Atmospheric Administration, USA) station, at Cross Village (45.6414°, −85.0142°) located less than 10 km from our site, for the years 1985 to 2022. Using the *rnoaa* R package (Chamberlain 2019; R Core Team 2021), we retrieved information on total precipitation and maximum, mean, and minimum air temperatures for each month. In addition to these four variables, we also calculated the Standardized Precipitation–Evapotranspiration Index (SPEI of Beguería and Vicente‐Serrano 2017). We converted all climate variables to monthly anomalies (i.e., *z*‐scores), with the reference value being the mean of all months (all the months of January, for example) from 1985 to 2022.

### 2.7 Sliding Window Analysis

To investigate whether climate contributes to the portfolio effect, we performed sliding window analyses on all five vital rates and tested for a climate‐by‐zone interaction. We used the *climwin* R package (Bailey and van de Pol 2016) to test a wide range of hypotheses for how climate variables influence vital rates. A detailed explanation on implementing the sliding window analysis with *climwin* can be found in van de Pol et al. (2016). This sliding window analysis mirrored the generalized linear mixed‐effect model for vital rates, by including size as a fixed effect for S2, G, and A. However, when compared with the vital rate models, our sliding window analysis fit models testing the fixed effects of climate, zone, and their interaction, using year as a random effect (e.g., surv_*t*1 ~ size_*t*0 + zone + climate: zone +1|year_*t*0). In these models, the climate‐by‐zone interaction tests the role of climate in driving asynchrony among zones, while the year random effect accounts for temporal variability not driven by climate. The candidate models included the five climate variables (precipitation, minimum, mean, and maximum temperatures, and SPEI) and 703 different time windows across a time range of 3 years before the census (30th of June) of the second year in a transition (namely, “year *t*1”). Given that the census is always the 30th of June, these were “absolute” climate windows (sensu van de Pol et al. 2016). We did not consider more than 3 years prior to the census because *C. pitcheri* is a short‐lived monocarpic perennial and a window occurring longer than 3 years before the vital rate response has only been found in a single, very long‐lived perennial plant species (Evers et al. 2021). The 703 different time windows were composed of all possible combinations of starting and ending months with the 37‐month time period that includes the census month and all 36 months prior to the census. Across each of these time windows, we computed the mean weather anomaly for each climate variable. For example, for a window of 3 months (e.g., from June to August), we computed the mean anomaly of precipitation of those 3 months. Then, for each window, we used the computed mean anomaly as a linear predictor in our vital rate model. Next, for each vital rate, we selected the model with the lowest Akaike Information Criterion corrected for small sample size (AICc, Burnham and Anderson 2002) across the baseline model and all candidate models for all five climate variables. While in principle, we could test two or more climate variables at a time, we tested one variable at a time for two reasons. First, SPEI already combines the information of precipitation and mean temperature and second, testing two variables at a time would increase the number of model fits by a factor of five. As a final step, we performed randomization tests to verify that the correlation found in the best model was not spurious, a realistic probability when testing hundreds of different models. During this randomization process, the years of the vital rate censuses were randomized 1000 times and the sliding window was rerun. In this case, any model support (AICc) found would be purely spurious. Running this process multiple times created a distribution of spurious AICc scores which was used to calculate the probability that the AICc found in the main sliding window analysis was due to chance. We compared all of our candidate models against this distribution of spurious AICc scores.

### 2.8 Does Climate–Zone Interaction Contribute to Portfolio Effects?

To test whether climate effects contributed to portfolio effects, we tested whether the asynchrony of vital rates among zones could be explained by different responses to climate. Specifically, we tested whether the asynchrony between Zone 1 and the remaining zones correlated with the climate‐by‐zone interaction in the climate models. To do so, we regressed the climate‐by‐zone interaction coefficients on the corresponding correlation between Zone 1 and the remaining zones. This analysis is justified because the climate‐by‐zone interactions compare the effect of climate in Zone 1 to the effect of climate in the other zones.

We performed analyses using R version 4.3.2 (R Core Team 2024). We fit generalized linear mixed models using lme4 (Bates et al. 2015), and we calculated SPEI using package SPEI (Beguería and Vicente‐Serrano 2017). To maximize the reproducibility of our analyses, our code repository uses package *renv* (Ushey and Wickham 2023).

## 3 Results

**FIGURE 3:**
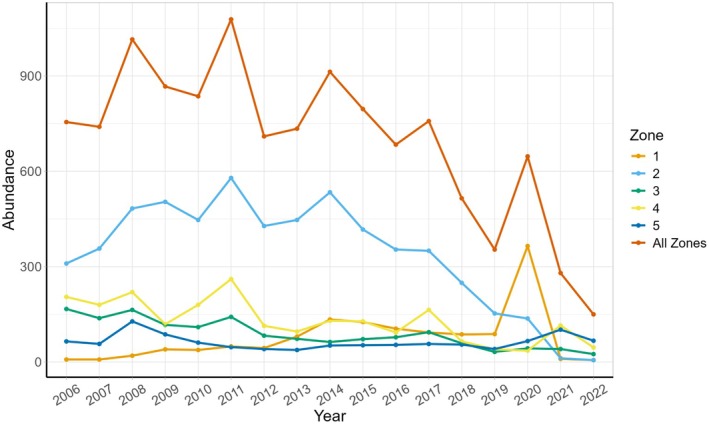
Total abundance of *Cirsium pitcheri* in the five zones and the entire plot over the 17‐year study period. Zones are 10 m × 40 m, and are explained in the main text and depicted in Figure 1. The peak in abundance in 2020 was primarily due to a bumper crop of seedlings in one small dune front area that was subsequently buried in 2021.

From 2006 to 2022, the annual abundance of *C. pitcheri* in our plot, including all stages—seedlings, vegetative plants, and flowering adults—ranged from a low of 150 to a high of 1078 plants for the entire plot (mean ± SD = 696 ± 248; Figure 3). Seedling abundance ranged from 21 to 465 (192 ± 143) and the mean number of flowering adults ranged from 10 to 133 (64 ± 33).

### 3.1 Vital Rates and Population Growth Rates Vary in Space and Time

**FIGURE 4:**
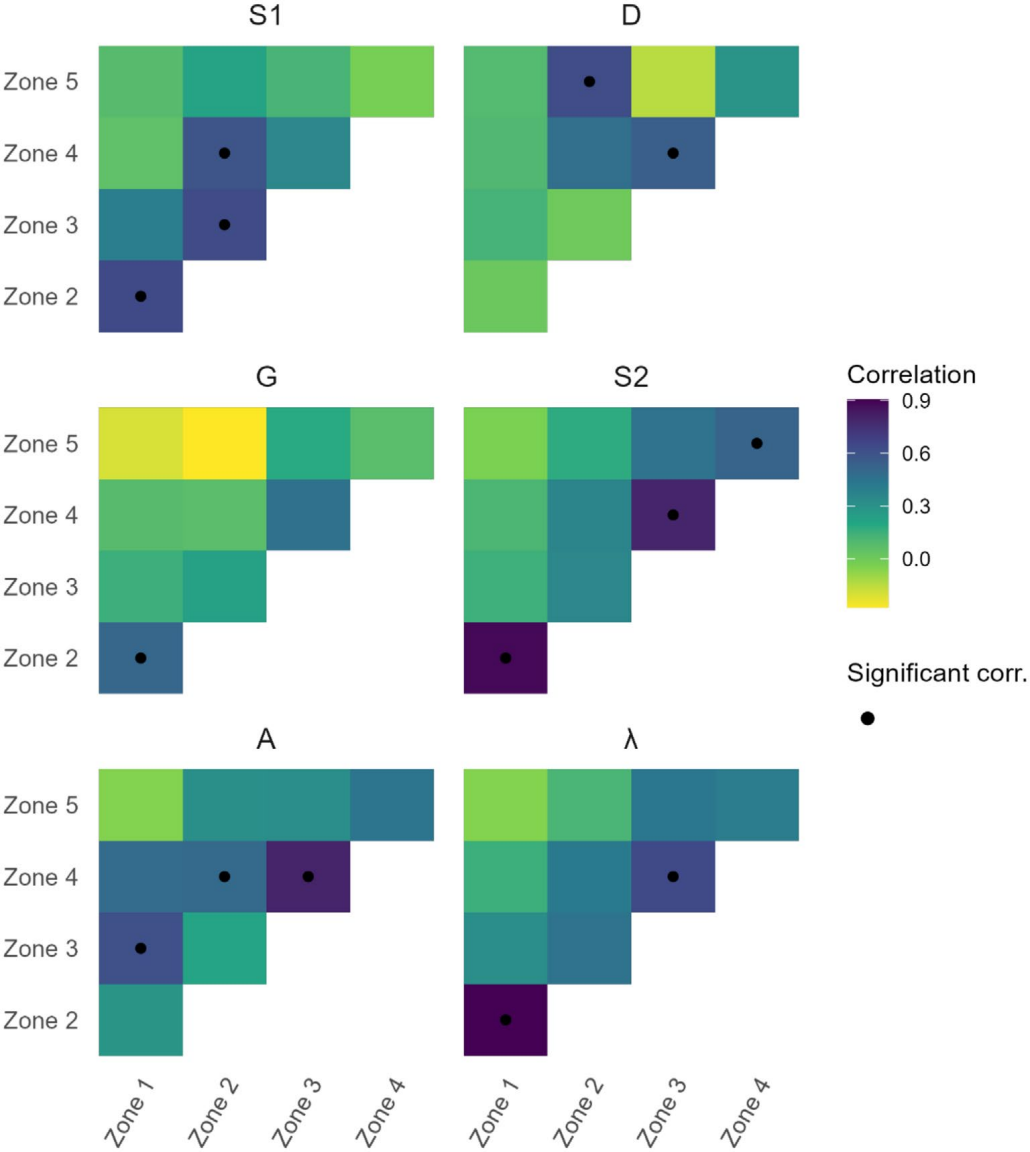
Vital rates and population growth rates tend to progressively desynchronize with distance. Correlation matrices show the correlation of vital rates across all combinations of the five zones. Lighter shades represent lower correlations, and darker shades represent higher correlations. Dots show significant correlations based on Pearson's product moment correlation. Panels refer to Seedling survivorship (S1); New plant size (D); Growth (G) for a mean‐sized plant; Plant survivorship (S2) for a mean‐sized plant; Probability of flowering (A) for a large (mean + 1 SD) plant; and Population growth rate (*λ*).

**FIGURE 5:**
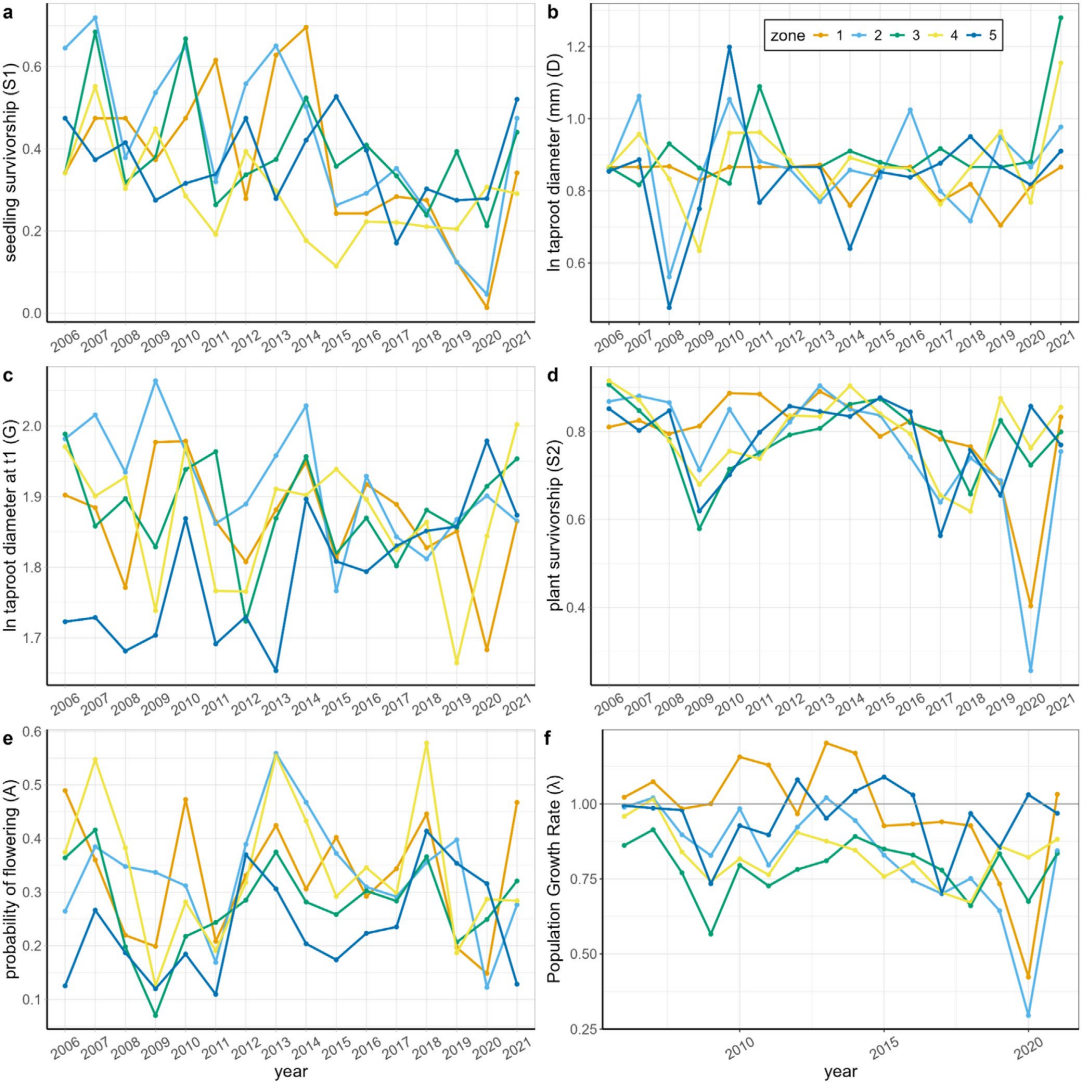
Vital rate models can show asynchronous variation in time and space; different zones responded in opposite directions to certain years. Lines and dots show the modeled mean estimates of vital rates in each year and zone. The color code for zones matches Figure 1; Zone 1 is closest to Lake Michigan. For the size‐independent vital rates (S1 and D), we fit this interaction as random intercepts. For the size‐dependent models (S2, G, and A), we included a random slope of size per year by zone. Then, we plotted the mean predictions of these models for each year and zone. For the models that included size, we plotted the mean prediction for individuals of a specific size: the mean size for S2 and G, and a size one standard deviation larger than the mean for A. We chose a larger size for visualizing models for A because flowering probability is uniformly low at small sizes, and variation in years and zones is only apparent when examining the larger‐sized plants. (a) Seedling survivorship probability (S1); (b) New plant size (D, ln taproot diameter (mm)); (c) Growth (G) shown as size in year *t*1 of a plant that was of mean size in year *t*0, ln(taproot diameter); (d) Survivorship probability (S2) of a mean‐sized plant; (e) Probability of flowering (A) for a large (mean + 1 SD) plant; and (f) population growth rate (*λ*).

Fluctuations in vital rates and population growth rates are significantly correlated for zones at close distance, almost exclusively, while the strength of correlations approaches zero as the distance between zones increases. Of our 14 significant correlations, 10 occur between directly adjacent zones (Figure 4). These patterns of correlation are exemplified by those of population growth rates. Population growth rates are strongly and significantly correlated between Zones 1 and 2 and between Zones 3 and 4, but their correlation between Zones 1 and 5 is indistinguishable from zero (Figure 4). Because the vital rates of Zones 1 and 5 are not correlated, these two zones occasionally fluctuate in opposite directions: for example, in year 2014 for seedling survival (Figure 5a), in 2019 for the size of new plants (Figure 5b), in 2009 for growth (Figure 5c), and in 2020 for adult survivorship (Figure 5d) and population growth rates (Figure 5f).

### 3.2 Small‐Scale Portfolio Effects

Our synchrony index was 0.35 and the PE was 2.01, indicating temporal variance among plots had substantial stabilizing effects on the variance of total population abundance. A synchrony index of 0.35 likely reflects that strong positive correlations among adjacent zones are offset by the weak or negative correlations among distant zones (Figure 4). A PE of 2.01 indicates that the observed variance in total population abundance is two times lower than what would be expected by the mean–variance scaling relationship alone (Equation 5).

### 3.3 Climate Effects Vary by Zone

**FIGURE 6:**
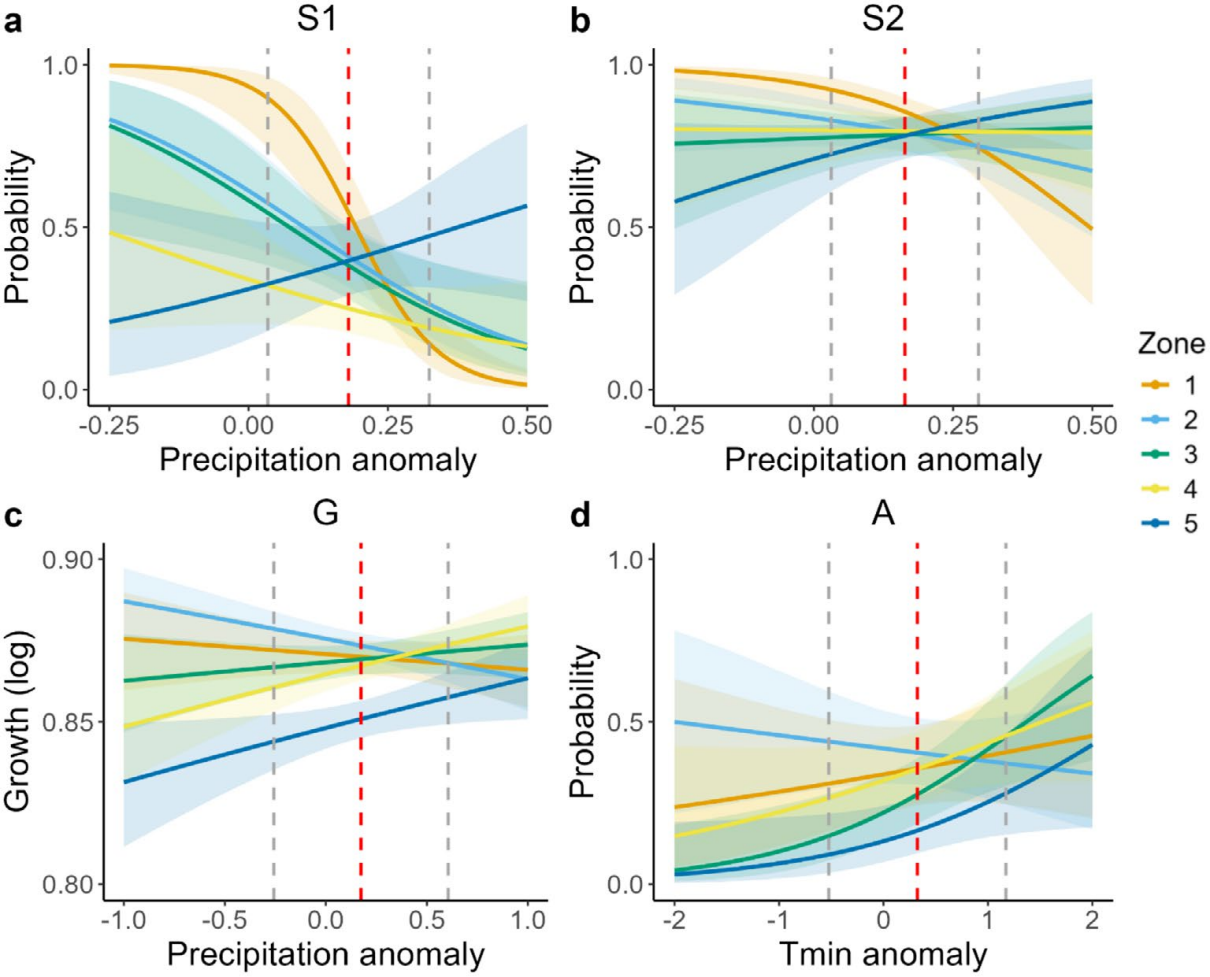
Spatial variation in modeled response to climate. The *x*‐axes for each panel are the scaled climate anomalies for the respective climate factor and window depicted in Figure 3. Zones 1 through 5 are described in Figure 1. (a) Seedling survivorship presented as a function of monthly precipitation during the 16‐month period prior to the census year; (b) Survivorship of a mean‐sized plant as a function of monthly precipitation during the 15 months prior to census; (c) Growth of a mean‐sized plant as a function of precipitation during 5 months prior to the census year; (d) Probability of flowering for a large (mean +1 SD) plant as a function of the minimum temperature during the single month of July of the year before flowering. Dashed vertical lines show mean (red) and ±1 SD (gray) values for respective climate anomalies during the 17‐year study period.

**FIGURE 7:**
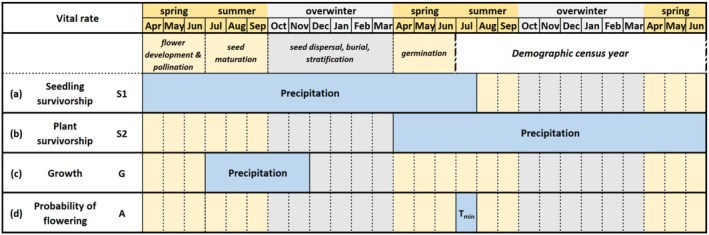
The significant climate driver and climate window influencing each vital rate, along with the timing of life cycle events and the timing of the demographic data collection (detailed results from the *climwin* analysis for each vital rate are provided in Figures S4–S7). The climate driver and climate window are shown in blue, the time window in which the demographic data are collected (June in year *t*0 to June in year *t*1) is shown in white, and the growing season and dormant season months prior to the demographic census year are shown in yellow and gray, respectively. The life cycle events are shown at the top and focus on processes that occur before seedlings are censused. (a) Seedling survivorship was best predicted by precipitation in a 16‐month period prior to the demographic census year. (b) Plant survivorship was best predicted by a 15‐month window including the demographic census year. (c) Growth of vegetative plants was best predicted by a 5‐month window in the year prior to the demographic census year. (d) Probability of flowering was best predicted by the minimum temperature in July 1 year before flowering. The span of 27 months shown includes all significant climate windows; the *climwin* analysis included 10 additional months not shown.

We found significant climate‐by‐zone interactions in all vital rates, with patterns that were strikingly similar for seedling survival (S1), adult survivorship (S2), and growth (G) (Figure 6 and Table 1). These three vital rates all responded to precipitation anomalies, although in different climate windows (Figure 7). Plants in Zone 1 responded negatively to higher precipitation, whereas those in Zone 5 responded positively to higher precipitation (Figure 6a–c). The size of new plants (D) was not explained by any of the climate factors; the best‐fit climate window failed the randomization test (Table S1). Tables comparing the five tested climate variables for each of the five vital rates are included in Tables S2–S6. Heat maps showing delta AICc for all tested windows for the most predictive climate variable are supplied for all vital rates except the size of new plants (D) in Figures S4–S7.

**TABLE 1:** Analysis of deviance tables of type II Wald *χ* ^2^ tests performed on the four vital rates with nonspurious climate predictors. Size at year one (D) had no probable nonspurious models. Tests were run using function Anova() from R package *car* (Fox and Weisberg 2019).

Vital rates and factors	*χ* ^2^	df	*p*
S1 (seedling survivorship)			
Climate	6.134	1	0.0133
Zone	66.409	4	**< 0.0001**
Climate:Zone	54.541	4	**< 0.0001**
S2 (plant survivorship)			
Size	0.0012	1	0.9727
Climate	0.6411	1	0.4233
Zone	5.7575	4	0.2180
Climate:Zone	44.8695	4	**< 0.0001**
G (Growth)			
Size	6818.9135	1	**< 0.0001**
Climate	0.4339	1	0.5100
Zone	124.8870	4	**< 0.0001**
Climate:Zone	74.8037	4	**< 0.0001**
A (Flowering probability)			
Size	708.0444	1	**< 0.0001**
Climate	0.3965	1	0.5289
Zone	21.7831	4	**0.0002**
Climate:Zone	33.0557	4	**< 0.0001**

*Note:* Bold values represent significant *p*‐values.

The *climwin* analysis identified the minimum temperature during the month of July in year *t*0 as the best climate predictor of the probability that a vegetative plant would flower the next year (year *t*1) (Figure 7d). While this temperature window did not explain the probability of flowering (there was no main effect of climate), we did find small but significant spatial (zone) effects as well as a significant climate‐by‐zone interaction (Figure 6d, Table 1). Plants in Zones 3 and 5 were significantly less likely to flower compared with those in Zone 1. Plants in Zone 3 were more likely to flower when the July temperature minimum was higher, whereas plants in the other zones did not respond to the July temperature minimum.

### 3.4 Portfolio Effects Are Partially Driven by a Climate‐by‐Zone Interaction

**FIGURE 8:**
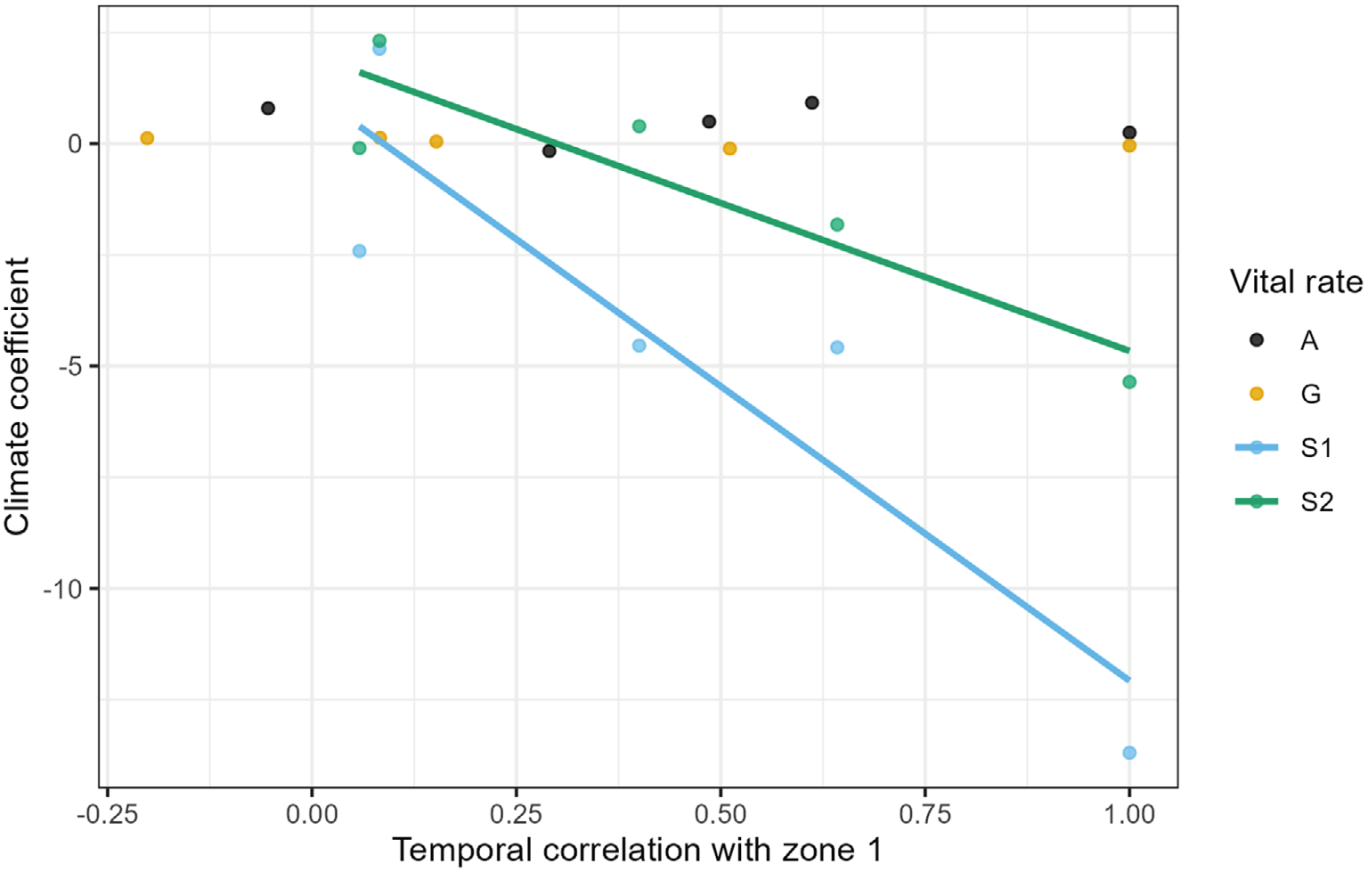
Climate‐by‐zone interaction explains asynchrony of adult and seedling survival among plots. Bivariate plot showing, for four vital rates, the coefficient of the effect of climate on each zone and the correlation coefficient between zone 1 and all remaining four zones. The colors refer to data points from four vital rates: Probability of flowering (A), Growth (G), Seedling survival (S1), and Plant Survival (S2). The lines show the average prediction of significant (*p* < 0.05) linear regressions.

In the adult and seedling survival models we found a significant association between the climate coefficients in each zone and the correlation between Zone 1 and all other zones (Figure 8). This association indicates that at least part of the asynchrony between Zone 1 and other zones is driven by contrasting responses to climate.

## 4 Discussion

Over the 17‐year monitoring period at Wilderness State Park, the overall abundance of *Cirsium pitcheri* has fluctuated widely, and has been declining in the most recent years. We found evidence that fluctuations in vital rates and population growth (*λ*) of *C. pitcheri* were correlated at short distances (zones within ~10 m) but were uncorrelated at farther distances (zones separated by 30–50 m). These independent fluctuations at farther distances were associated with the significant climate‐by‐zone interactions we found using sliding windows analyses. In other words, asynchrony among populations was driven by differential responses of demography to climate.

### 4.1 Climate Predictors With Non‐Obvious Windows

Most studies of climate impacts on demographic variation assume that growing season conditions are the most important (e.g., Abbott, Doak, and DeMarche 2017). However, lagged climate conditions may be important drivers of ecological responses (Czachura and Miller 2020; Evers et al. 2021). In our analysis, the climate windows differed for the three vital rates whose variation was predicted by precipitation anomalies. These differences included immediate effects on plant survivorship, which was affected by precipitation during the transition year, and lagged effects of precipitation on plant growth and seedling survivorship. A more intuitive (and more narrow) climate window best predicted the likelihood of flowering in our plot: minimum temperature in July of year_*t*0 predicting flowering in year_*t*1.

### 4.2 Climate‐by‐Zone Interactions

The climate‐by‐zone interaction patterns for the three vital rates responding to precipitation anomalies are consistent with the mechanism that storms have negative effects on exposed plants on the foredunes (Zones 1 and 2), but that the increased precipitation that these storms bring has mostly positive effects on the growth and survivorship of more protected plants on the backdunes (Zone 5). Unfortunately, we cannot definitively test this idea because data on storm intensity (e.g., wind and wave data) are mostly unavailable for the NOAA station closest to our site. The effects on plant survivorship are straightforward: storms kill plants by sand burial in the foredunes and provide added water resources in the backdune, enhancing survival there. For plants that manage to survive in the lake‐facing zones (Zones 1 and 2) despite being buried, energy reserves would be committed to emergence upward and not taproot growth (Maun and Perumal 1999), explaining the precipitation‐by‐zone interaction for growth.

For seedlings, the 16‐month window in which precipitation influences survivorship includes the period of flower development for their mothers, seed maturation on the mother plant, seed dispersal, seed burial, and seed germination (Figure 7). For the lake‐facing zones, storm‐mediated sand movement might cause maternal plants that are buried to commit fewer resources to developing seeds. Previous work has shown that seedling success is a function of seed size for *C. pitcheri* (Chen and Maun 1999; Hamzé and Jolls 2000). Further, those seeds, when dispersed, would be buried more deeply, requiring more of the stored energy to reach the sand surface to begin life as a seedling (Chen and Maun 1999). These effects would lead to fewer belowground resources available for seedling survival, explaining the precipitation‐by‐zone interaction effect for that vital rate. Alternatively, years of high precipitation could promote germination of seedlings in what are typically dry microsites on the front of the dune (Zones 1 and 2) that would not normally support them; these seedlings would eventually die. However, the long climate window associated with seedling survival makes that explanation less likely.

In addition to the effects of storms on plants through sand burial, there are other possible mechanisms by which precipitation could impact plant demographics differently across zones. For example, increases in precipitation could negatively affect seedlings in Zones 1 and 2 by positively influencing the abundance of their competitors (Suttle et al. 2007). We deem this mechanism less likely because dune ecosystems like ours have few competitors in the early successional stages associated with Zones 1 and 2, and more competitors in the backdunes (Doing 1985). However, more work is needed in this area. We know very little about the effects of competition on *C. pitcheri* , especially beyond the vulnerable seedling stage; Rand et al. (2015) found that proximity to neighbors increased the likelihood of germination in drier habitats but did not influence seedling survival.

The probability of flowering was weakly predicted by temperature during the summer of the year before flowering (minimum temperature of July in year *t*0). Across all years (i.e., independent of climate), plants in Zone 3 and especially Zone 5 were the least likely to flower. The backdune environment of Zone 5 is typically the hottest zone, where plants have the lowest growth rates overall. Plants in this zone may take longer to reach size thresholds and are therefore less likely to flower (Metcalf et al. 2003; Priemer and Girdler 2024). For plants in Zone 3, which is the highest point on the dune facing the lake, higher July temperatures in year *t*0 lead to a higher likelihood of flowering the following year for plants in Zone 3 compared with cooler years. Warmer minimum temperatures may interact with air movement in those lake‐facing dune‐top microsites and lead to increased evapotranspiration, although why that would cause growth and a higher likelihood of flowering is unclear. Higher rates of evapotranspiration might stress competitor plants and indirectly allow more growth of large *C. pitcheri* individuals with well‐developed taproots. Since size is a strong predictor of flowering, these Zone 3 plants might then be able to grow enough to trigger flowering. Clearly, more research on competitive interactions in this species is warranted. Future analysis will need to parse the influence of plant age, size, and growth history to explain these intriguing patterns of reproduction.

### 4.3 Linking Climate–Zone Interactions to Portfolio Effects

The spatial asynchrony in *C. pitcheri* response to climate anomalies can explain the PE we found using population abundance data. Small scale variation across zones in *C. pitcheri* demographic response to climate variation dampens variability in abundance of the entire population by a factor of two. Without the asynchrony across the dune subpopulations, the already considerable population fluctuations would be larger, and the overall decline in population abundance observed over the 17‐year study period might have been even more pronounced. Indeed, a similar population in Wisconsin that was entirely in a dune‐facing habitat like our Zones 1 and 2 was completely extirpated during the 2019–2020 transition (Vitt et al. 2024), the same year our population had its most marked decline.

Previous studies found similar results, but at somewhat larger spatial scales. Oldfather and Ackerly (2018) described 16 populations across the elevational distribution of an alpine plant ( *Ivesia lycopodioides var. scandularis* ) in the xeric White Mountain range in eastern California. They found complex relationships between climate variables and population dynamics, which resulted in overall stable population dynamics across the species' distribution (~50 linear km and 600 m elevation). Dibner et al. (2019) quantified vital rate variation as well as *λ*s in a dryland endemic plant, *Yermo xanthocephalus* , sampled in 384,100 m^2^ plots in a grid covering 5 km^2^ in Wyoming. They found that asynchronous dynamics acted as a stabilizing mechanism for this rare species, with variation in response to growing season precipitation driving asynchrony in population abundance. Abbott, Doak, and DeMarche (2017) investigated a similar question in the rare alpine perennial plant, *Saussurea weberi* , in Colorado. They quantified population abundance in 49 1 m^2^ plots over a 5 ha area, finding that spatial asynchrony strongly reduced the variation in abundance through time for the entire population. Similar to our study, they found a significant plot by climate interaction whereby only synchronous plots showed an association with June temperatures.

Here, we demonstrate portfolio effects at an even smaller spatial scale—tens of meters. Hence, our results open up the possibility that differences in the response of populations to climate may vary at any scale and buffer against temporal variation. With our demographic data, we were also able to separately examine the different vital rates that contribute to the population growth of *C. pitcheri* and thus more finely discern how portfolio effects across small distances played out through spatial and climate interactions.

However, conclusions drawn from a single population with five replicate subpopulations might not generalize to other *C. pitcheri* range locations. Future research will need to clarify whether these portfolio effects are common across the range of *C. pitcheri* . Notably, the Wisconsin *C. pitcheri* population, which did not host a backdune subpopulation, was recently extirpated (Vitt et al. 2024). We hypothesize that the diversity of environments occupied by each *C. pitcheri* population might suggest the extent and relevance of the portfolio effects they are experiencing. To generalize from our small‐scale study, we must understand how the vital rates and population growth of other populations of *C. pitcheri* and other dune endemic species respond to climate drivers and dune microhabitats at different sites. For *C. pitcheri* , we can draw on several other demographic studies at sites that span its latitudinal range (Halsey et al. 2017; Havens et al. 2012; Jolls et al. 2019; McEachern et al. 1994), incorporating site‐specific climate variables to discover whether stabilizing portfolio effects are present in other populations and at other scales.

For *C. pitcheri* , we hypothesize that the presence of small‐scale stabilizing portfolio effects may mean that the “key” habitat is not always the iconic open sand dune microsites one finds in the U.S. Fish and Wildlife Service species abstract (USFWS, n.d.). Instead, this species and perhaps other dune “specialists” may require a mosaic of dune successional sites. Such ecosystem complexity may be critical for many populations faced with increasing climate variability (Abbott, Doak, and DeMarche 2017).

## Author Contributions

**E. Binney Girdler:** conceptualization (equal), data curation (lead), formal analysis (equal), funding acquisition (equal), investigation (equal), methodology (equal), project administration (equal), resources (lead), software (equal), supervision (lead), validation (lead), visualization (equal), writing – original draft (lead), writing – review and editing (lead). **Tiffany M. Knight:** conceptualization (equal), formal analysis (supporting), funding acquisition (equal), methodology (supporting), project administration (equal), resources (supporting), writing – original draft (equal), writing – review and editing (equal). **Sanne M. Evers:** conceptualization (supporting), formal analysis (equal), investigation (supporting), methodology (equal), software (equal), writing – original draft (supporting), writing – review and editing (supporting). **Aldo Compagnoni:** conceptualization (supporting), formal analysis (equal), investigation (equal), methodology (equal), software (supporting), visualization (supporting), writing – original draft (equal), writing – review and editing (equal). **Roxanne Leberger:** formal analysis (supporting), visualization (supporting). **Samara I. Hamzé:** data curation (supporting), investigation (supporting), methodology (supporting). **Julie E. Marik:** data curation (equal), investigation (supporting). **Claudia L. Jolls:** conceptualization (supporting), funding acquisition (supporting), investigation (supporting), methodology (supporting), project administration (supporting), writing – review and editing (supporting).

## Conflicts of Interest

The authors declare no conflicts of interest.

## Supporting information


**Data S1:** ece372080‐sup‐0001‐DataS1.pdf.

## Data Availability

Data and code used in this manuscript are available as a “private‐for‐review” link on Figshare: https://figshare.com/s/4df3e6955d86021238a2. Upon acceptance, data, and R scripts will be published via a Figshare DOI.
