# Supplementary material for: Small‐Scale Stabilizing Effect of Asynchrony in Vital Rate Responses to Climate in an Imperiled Dune Thistle
Source: Ecol Evol. 2025 Aug 31;15(9):e72080. doi: 10.1002/ece3.72080 (PMC12399319; doi:10.1002/ece3.72080)
Supplement: Supplementary file 1 — Data S1: ece372080‐sup‐0001‐DataS1.pdf. [file ECE3-15-e72080-s001.pdf]

Small-scale stabilizing effect of asynchrony in vital rate responses to climate in an imperiled dune thistle.

Appendix S1; Spatio-temporal and size-dependent variation in vital rates, and additional sliding window results

E. Binney Girdler, Tiffany M. Knight, Sanne M. Evers, Aldo Compagnoni, Roxanne Leberger, Julie E. Marik, Samara I. Hamzé, Claudia L. Jolls

Contents

Vital rate model predictions **2**

Summary tables **4**

Vital rate model summary **6**

Seedling survival (S1) 7

Size distribution of new vegetative plants (D) 8

Adult survival (S2) 9

Growth or changes in size (G) 10

Flower probability of adult plants (A) 12

Vital rate model predictions

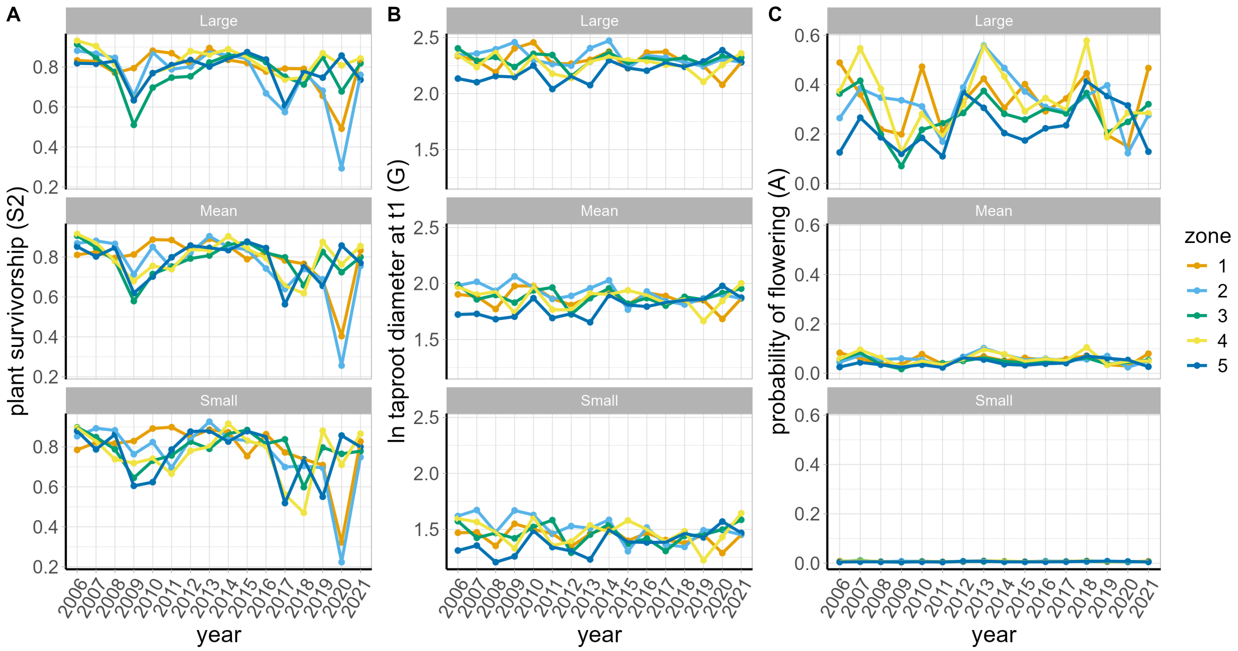

Figure S1: Variation in size-dependent vital rates through time and space. (A) Plant survivorship for small (mean - 1 sd), mean, and large (mean + 1 sd) plants; (B) Growth, shown as the estimated size of a plant in year t1 that started as a small (mean - 1 sd), mean, and large (mean + 1 sd) plant; (C) Probability of flowering for small (mean - 1 sd), mean, and large (mean + 1 sd) plants.

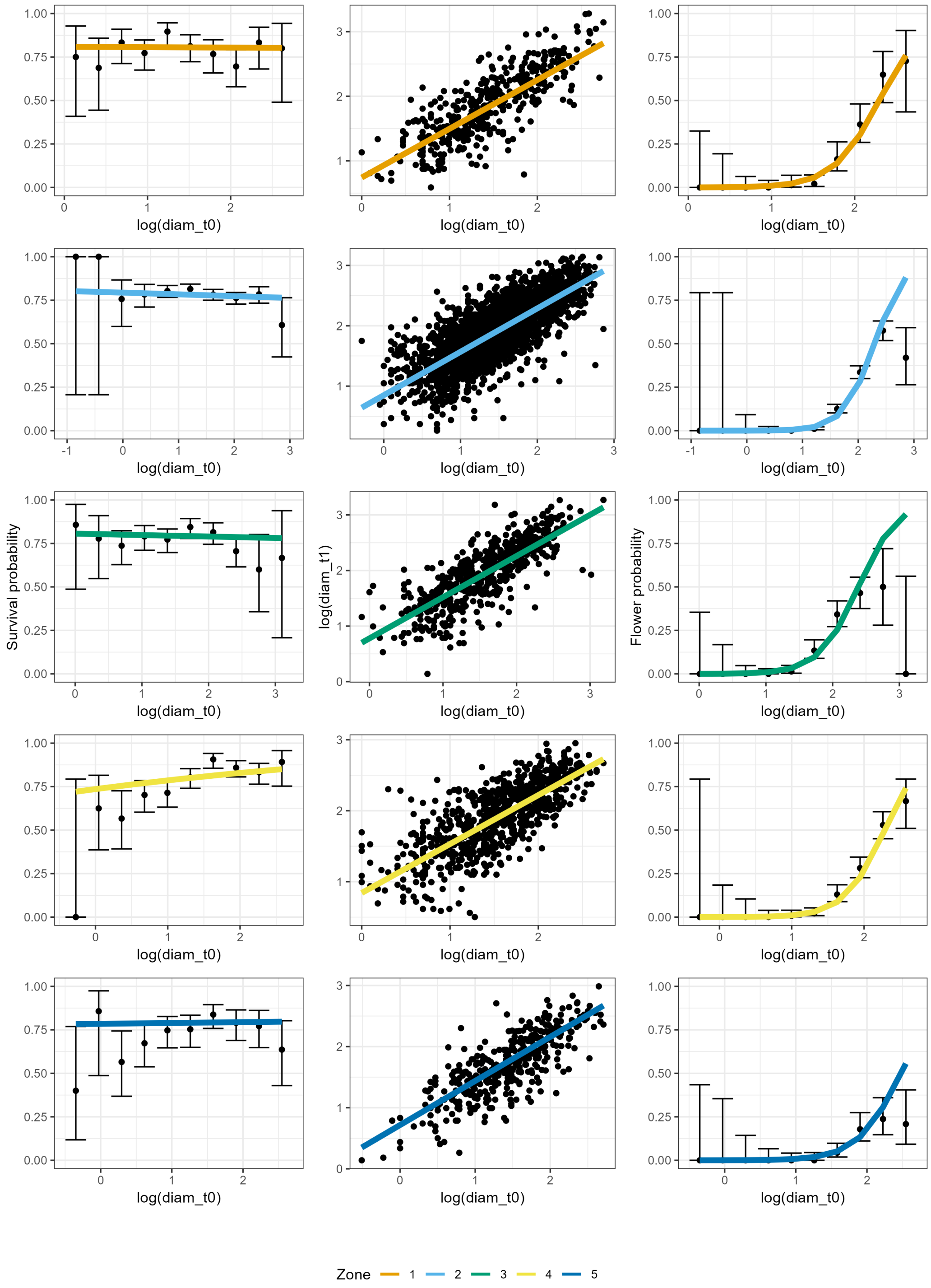

Figure S2: Zone-specific vital rate data and model predictions as a function of size for (A) plant survivorship; S2; (B) growth, G; and (C) flowering probability, A. Data and predictions combine all 17 years of data collected across each zone. Survival and flower probability data are proportions binned across ten equally spaced size intervals. The error bars around the proportion data are the 95% confidence intervals calculated using the Wilson score interval. Zone 1 is nearest the lake, and zone 5 is on the back dune adjacent to the forest.

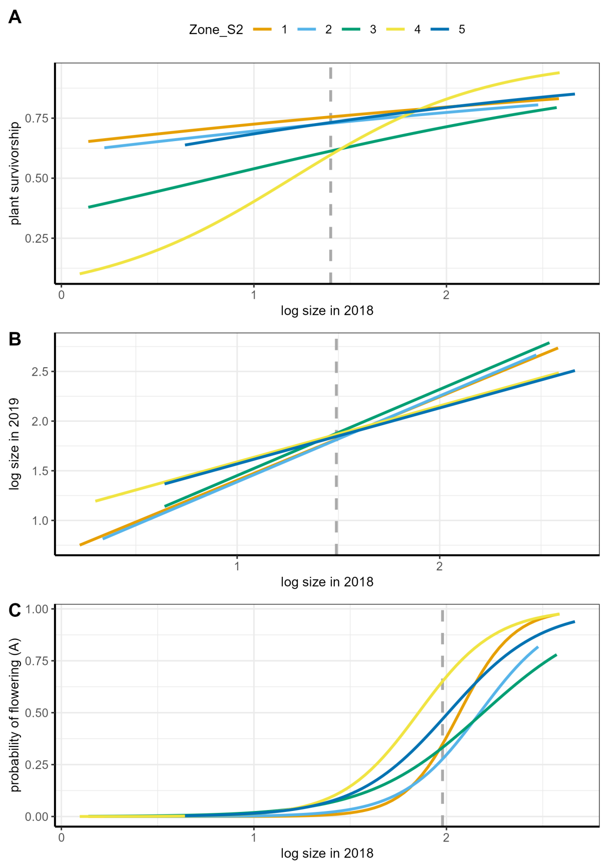

Figure S3: Vital rate model predictions for a single year show zone-specific size dependence of (A) plant survivorship; S2; (B) growth, G; and (C) flowering probability, A, in 2018. Dashed lines in (A) and (B) show predictions referred to mean plant size; in (C) the dashed line shows large plant size (mean + 1 sd). Colors refer to the zone-specific predictions. Zone 1 is nearest the lake, and zone 5 is on the back dune adjacent to the forest.

Summary tables

Below are the summary tables for for the randomization tests, and for each of the vital rates. The table on randomization tests lists the probabilities of the correlations found by the best models of each vital rate to be spurious correlations. These probabilities were tested using the randomization analysis of the R-package `climwin`. The tables on vital rates shows the best time window for each of the tested climate variables.

Table S1: Summary of the randomization results for the five vital rates. For each vital rate, the best climate variable and time window (WindowOpen/WindowClose) is listed. The probability that the correlation found in these windows is due to a spurrious correlation was tested using the randomization process.

Vital rate (Abbreviation)	Climate variable	WindowOpen	WindowClose	# Randomizations	P-value
Seedling survival (S1)	ppt	26	11	1000	0.002
Seedling size (D)	ppt	27	11	1000	0.925
Adult survival (S2)	ppt	14	0	1000	<0.001
Growth (G)	ppt	23	19	1000	0.005
Flower probability (A)	tmin	11	11	1000	<0.001

Table S2: Summary of the climate driver combinations for the sliding window analysis of the seedling survival probability of *Cirsium pitcheri*. The first column is the climate variable tested, the ModelAICc column lists the AICc of the model with the lowest AICc of that climate variable, the DeltaAICc columns shows the difference between the model with the lowest AICc, and the baseline model that does not include any climate $[S1 \sim zone + (1|year_{t0})]$. The last two columns show the opening and closing month (i.e., beginning and end of the time window). When the open and close windows are the same, this means the specific time window spans that one month.

Climate variables	ModelAICc	ΔAICc	WindowOpen	WindowClose
tmean	3535.839	-40.94	29	29
ppt	3518.726	-58.05	26	11
tmax	3530.800	-45.97	29	29
tmin	3539.326	-37.45	31	31
SPEI	3536.622	-40.15	1	1

Table S3: Summary of the climate driver combinations for the sliding window analysis of the size distribution of seedlings transitioning to adult plants of *Cirsium pitcheri*. The first column is the climate variable tested, the ModelAICc column lists the AICc of the model with the lowest AICc of that climate variable, the DeltaAICc columns shows the difference between the model with the lowest AICc, and the baseline model that does not include any climate $[D \sim zone + (1|year_{t0})]$. The last two columns show the opening and closing month (i.e., beginning and end of the time window). When the open and close windows are the same, this means the specific time window spans that one month.

Climate variables	ModelAICc	ΔAICc	WindowOpen	WindowClose
tmean	556.7315	-8.47	13	13
ppt	539.6190	-25.59	27	11
tmax	556.9067	-8.30	13	13
tmin	560.3154	-4.89	13	13
SPEI	562.4250	-2.78	12	12

Table S4: Summary of the climate driver combinations for the sliding window analysis of the adult survival probability of *Cirsium pitcheri*. The first column is the climate variable tested, the ModelAICc column lists the AICc of the model with the lowest AICc of that climate variable, the DeltaAICc columns shows the difference between the model with the lowest AICc, and the baseline model that does not include any climate $[S2 \sim size_{t0} + zone + (1|year_{t0})]$. The last two columns show the opening and closing month (i.e., beginning and end of the time window). When the open and close windows are the same, this means the specific time window spans that one month.

Climate variables	ModelAICc	ΔAICc	WindowOpen	WindowClose
tmean	5812.621	-23.11	19	19
ppt	5796.929	-38.80	14	0
tmax	5816.768	-18.96	19	19
tmin	5806.727	-29.01	20	19
SPEI	5809.163	-26.57	17	0

Table S5: Summary of the climate driver combinations for the sliding window analysis of the growth of adult plants of *Cirsium pitcheri*. The first column is the climate variable tested, the ModelAICc column lists the AICc of the model with the lowest AICc of that climate variable, the DeltaAICc columns shows the difference between the model with the lowest AICc, and the baseline model that does not include any climate $[G \sim size_{t0} + zone + (1|year_{t0})]$. The last two columns show the opening and closing month (i.e., beginning and end of the time window). When the open and close windows are the same, this means the specific time window spans that one month.

Climate variables	ModelAICc	ΔAICc	WindowOpen	WindowClose
tmean	2594.057	-8.43	11	9
ppt	2561.395	-41.09	23	19
tmax	2600.451	-2.03	36	32
tmin	2578.649	-23.84	11	9
SPEI	2595.924	-6.56	20	10

Table S6: Summary of the climate driver combinations for the sliding window analysis of the flower probability of adult plants of *Cirsium pitcheri*. The first column is the climate variable tested, the ModelAICc column lists the AICc of the model with the lowest AICc of that climate variable, the DeltaAICc columns shows the difference between the model with the lowest AICc, and the baseline model that does not include any climate $[A \sim size_{t0} + zone + (1|year_{t0})]$. The last two columns show the opening and closing month (i.e., beginning and end of the time window). When the open and close windows are the same, this means the specific time window spans that one month.

Climate variables	ModelAICc	ΔAICc	WindowOpen	WindowClose
tmean	2428.666	-23.81	11	11
ppt	2439.191	-13.29	29	29
tmax	2433.612	-18.87	11	11
tmin	2425.916	-26.56	11	11
SPEI	2447.536	-4.94	33	33

Vital rate model summary

Below we summarize the sliding window analysis for the best climate variable for each vital rate, as well as the summary of the models, using the best time window. The triangle graphs show the ΔAICc of the models using the different time windows with opening and closing months on the y- and x-axis, respectively. This means that the area in the upper left corner of each of the graphs shows the windows spanning most or all of the total time range. The windows closer to the perpendicular are short windows, with the window in the lower left corner being the window with only the first month.

Seedling survival (S1)

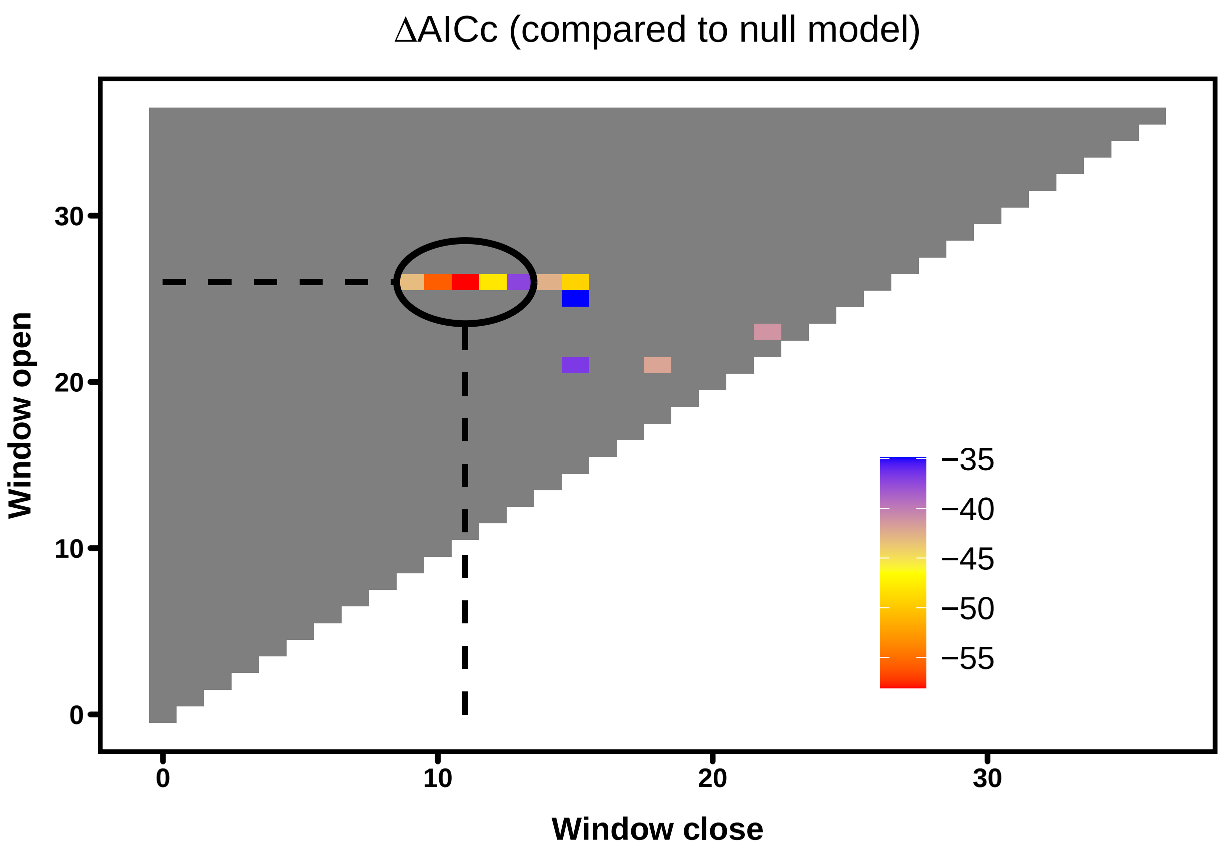

Figure S4: Heat map showing the deltaAICc for the model with precipitation in that particular window and the baseline model that does not contain climate. Windows without a value (grey) had a p-value > 0.05 when tested for spurious correlations.

```
## Generalized linear mixed model fit by maximum likelihood (Laplace
##   Approximation) [glmerMod]
##  Family: binomial  ( logit )
## Formula: yvar ~ climate + zone + (1 | year_t0) + climate:zone
##    Data: modeldat
##
##      AIC      BIC   logLik deviance df.resid
##   3518.6   3585.3  -1748.3   3496.6     3147
##
## Scaled residuals:
##     Min      1Q  Median      3Q     Max
## -2.7125 -0.7023 -0.4470  0.9107  7.8331
##
## Random effects:
##  Groups  Name        Variance Std.Dev.
##  year_t0 (Intercept) 0.5924   0.7697
## Number of obs: 3158, groups:  year_t0, 16
##
```

```
## Fixed effects:
##                Estimate Std. Error z value Pr(>|z|)
## (Intercept)      2.6394     0.6400   4.124 3.72e-05 ***
## climate       -13.6950     2.6059  -5.255 1.48e-07 ***
## zone2          -2.1823     0.5592  -3.902 9.52e-05 ***
## zone3          -2.3065     0.5803  -3.975 7.04e-05 ***
## zone4          -3.3060     0.5676  -5.825 5.72e-09 ***
## zone5          -3.4418     0.6063  -5.676 1.38e-08 ***
## climate:zone2   9.1136     2.2887   3.982 6.83e-05 ***
## climate:zone3   9.1543     2.4215   3.780 0.000157 ***
## climate:zone4  11.2807     2.3600   4.780 1.75e-06 ***
## climate:zone5  15.8302     2.3810   6.649 2.96e-11 ***
## ---
## Signif. codes:  0 '***' 0.001 '**' 0.01 '*' 0.05 '.' 0.1 ' ' 1
```

Size distribution of new vegetative plants (D)

No probable non-spurious models found.

Adult survival (S2)

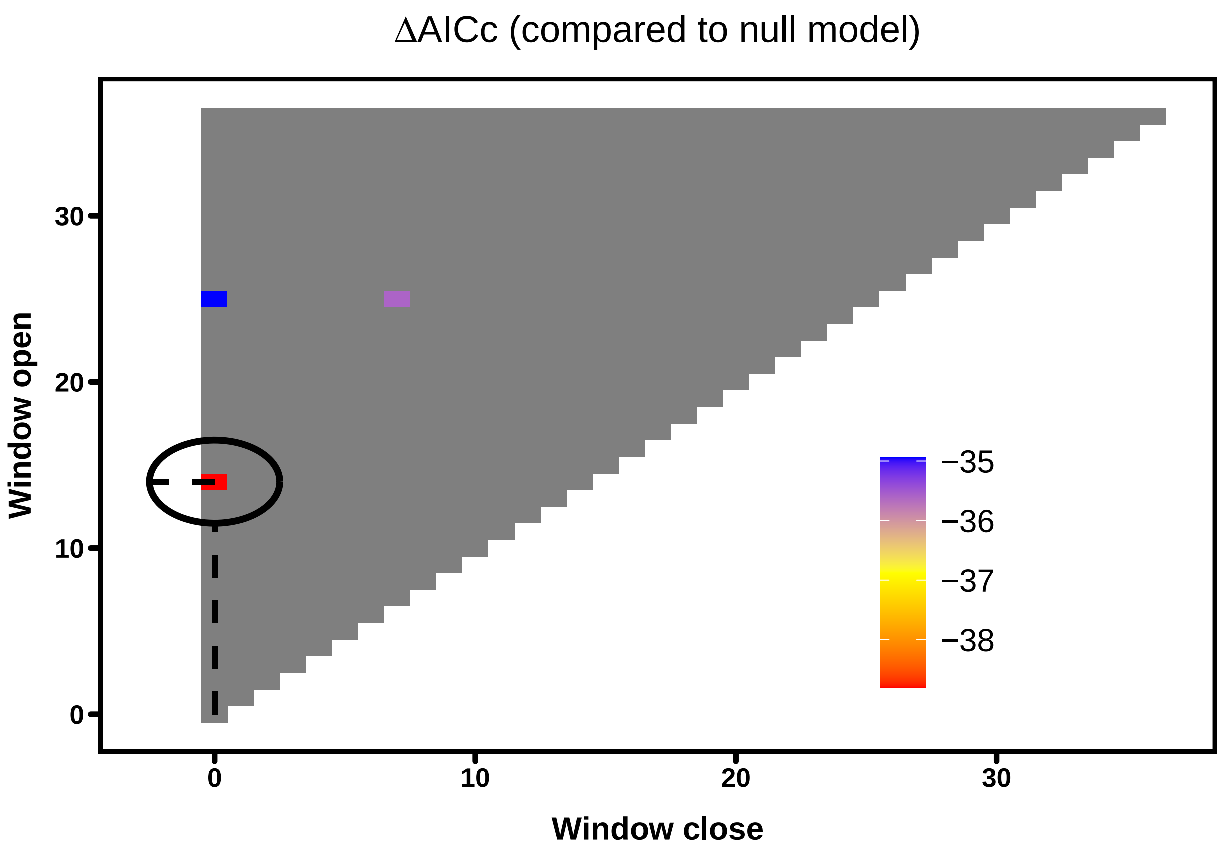

Figure S5: Heat map showing the deltaAICc for the model with precipitation in that particular window and the baseline model that does not contain climate. Windows without a value (grey) had a p-value > 0.05 when tested for spurious correlations.

```
## Generalized linear mixed model fit by maximum likelihood (Laplace
##   Approximation) [glmerMod]
##  Family: binomial  ( logit )
## Formula: yvar ~ size_t0 + climate + zone + (1 | year_t0) + climate:zone
##    Data: modeldat
##
##      AIC      BIC   logLik deviance df.resid
##   5796.9   5877.1  -2886.4   5772.9     5880
##
## Scaled residuals:
##     Min      1Q  Median      3Q     Max
## -4.8922  0.3251  0.4121  0.5427  1.3608
##
## Random effects:
##  Groups  Name        Variance Std.Dev.
##  year_t0 (Intercept) 0.3844   0.62
## Number of obs: 5892, groups:  year_t0, 16
##
```

```
## Fixed effects:
##                Estimate Std. Error z value Pr(>|z|)
## (Intercept)    2.650795   0.387830   6.835 8.20e-12 ***
## size_t0        0.001948   0.056857   0.034 0.972665
## climate       -5.358795   1.608446  -3.332 0.000863 ***
## zone2         -1.021252   0.283749  -3.599 0.000319 ***
## zone3         -1.420843   0.301579  -4.711 2.46e-06 ***
## zone4         -1.277435   0.305755  -4.178 2.94e-05 ***
## zone5         -1.757015   0.311084  -5.648 1.62e-08 ***
## climate:zone2  3.539233   1.149841   3.078 0.002084 **
## climate:zone3  5.752570   1.274884   4.512 6.41e-06 ***
## climate:zone4  5.261367   1.285044   4.094 4.23e-05 ***
## climate:zone5  7.676460   1.361749   5.637 1.73e-08 ***
## ---
## Signif. codes:  0 '***' 0.001 '**' 0.01 '*' 0.05 '.' 0.1 ' ' 1
```

Growth or changes in size (G)

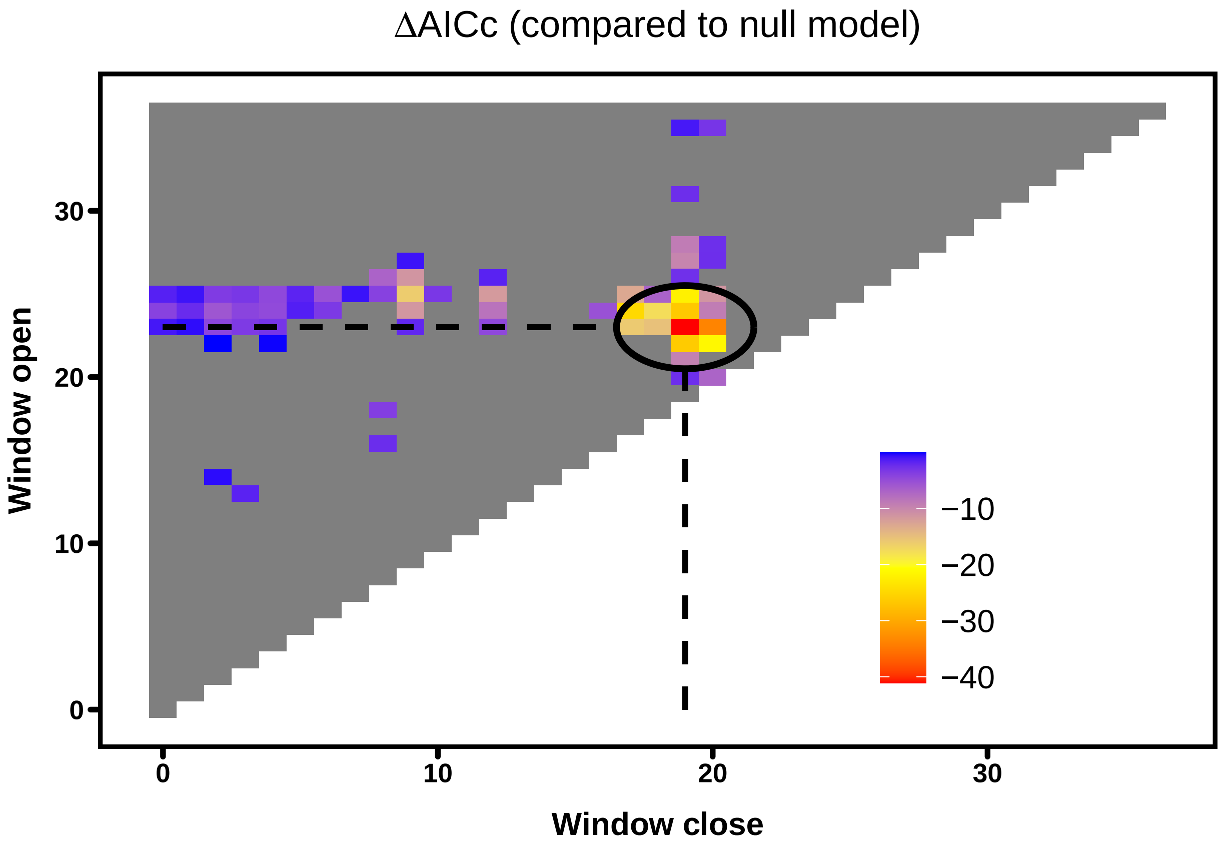

Figure S6: Heat map showing the deltaAICc for the model with precipitation in that particular window and the baseline model that does not contain climate. Windows without a value (grey) had a p-value > 0.05 when tested for spurious correlations.

```
## Linear mixed model fit by REML ['lmerMod']
```

```
## Formula: yvar ~ size_t0 + climate + zone + (1 | year_t0) + climate:zone
##    Data: modeldat
##
## REML criterion at convergence: 2535.3
##
## Scaled residuals:
##     Min      1Q  Median      3Q     Max
## -4.7973 -0.6022  0.0365  0.6564  3.7263
##
## Random effects:
##  Groups   Name        Variance Std.Dev.
##  year_t0  (Intercept) 0.004072 0.06382
##  Residual             0.102950 0.32086
## Number of obs: 4333, groups:  year_t0, 16
##
## Fixed effects:
##               Estimate Std. Error t value
## (Intercept)    0.838183   0.028572  29.335
## size_t0        0.718284   0.008698  82.577
## climate       -0.042317   0.054719  -0.773
## zone2          0.042550   0.020174   2.109
## zone3         -0.022480   0.023915  -0.940
## zone4         -0.053916   0.023085  -2.336
## zone5         -0.188606   0.025672  -7.347
## climate:zone2 -0.067913   0.039392  -1.724
## climate:zone3  0.091057   0.048581   1.874
## climate:zone4  0.174278   0.048407   3.600
## climate:zone5  0.166345   0.053567   3.105
```

Flower probability of adult plants (A)

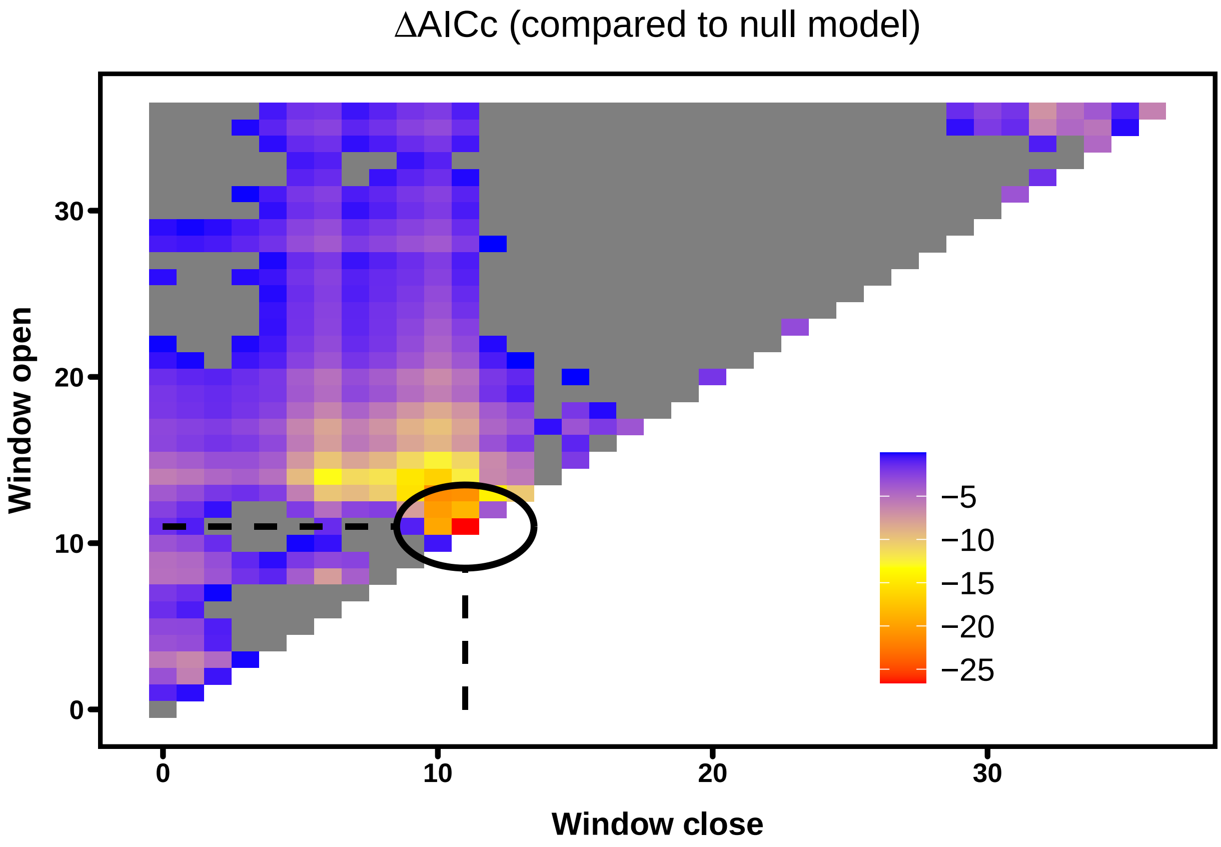

Figure S7: Heat map showing the deltaAICc for the model with minimum temperature in that particular window and the baseline model that does not contain climate. Windows without a value (grey) had a p-value > 0.05 when tested for spurious correlations.

```
## Generalized linear mixed model fit by maximum likelihood (Laplace
##   Approximation) [glmerMod]
##  Family: binomial  ( logit )
## Formula: yvar ~ size_t0 + climate + zone + (1 | year_t0) + climate:zone
##    Data: modeldat
##
##      AIC      BIC   logLik deviance df.resid
##   2425.8   2502.3  -1200.9   2401.8     4315
##
## Scaled residuals:
##     Min      1Q  Median      3Q     Max
## -6.9393 -0.3081 -0.1078 -0.0265 11.2989
##
## Random effects:
##  Groups  Name        Variance Std.Dev.
##  year_t0 (Intercept) 0.6158   0.7847
## Number of obs: 4327, groups:  year_t0, 16
##
```

```
## Fixed effects:
##                Estimate Std. Error z value Pr(>|z|)    
## (Intercept)   -9.95253    0.45381 -21.931  < 2e-16 ***
## size_t0        4.50770    0.16940  26.609  < 2e-16 ***
## climate        0.24862    0.34128   0.729 0.466303    
## zone2          0.34037    0.22264   1.529 0.126317    
## zone3         -0.59122    0.28350  -2.085 0.037028 *  
## zone4         -0.08484    0.25362  -0.335 0.737986    
## zone5         -1.20742    0.36184  -3.337 0.000847 ***
## climate:zone2 -0.41315    0.25922  -1.594 0.110968    
## climate:zone3  0.67328    0.31513   2.137 0.032638 *  
## climate:zone4  0.24827    0.29000   0.856 0.391934    
## climate:zone5  0.54861    0.38955   1.408 0.159036    
## ---
## Signif. codes:  0 '***' 0.001 '**' 0.01 '*' 0.05 '.' 0.1 ' ' 1
```
